# Degradation of biological macromolecules supports uncultured microbial populations in Guaymas Basin hydrothermal sediments

**DOI:** 10.1038/s41396-021-01026-5

**Published:** 2021-06-10

**Authors:** Sherlynette Pérez Castro, Mikayla A. Borton, Kathleen Regan, Isabella Hrabe de Angelis, Kelly C. Wrighton, Andreas P. Teske, Marc Strous, S. Emil Ruff

**Affiliations:** 1 Ecosystems Center and Bay Paul Center, Marine Biological Laboratory, Woods Hole, MA USA; 2 Soil and Crop Sciences, Colorado State University, Fort Collins, CO USA; 3 Department of Geosciences, University of Calgary, Calgary, AB Canada; 4 Department of Marine Sciences, University of North Carolina, Chapel Hill, NC USA; 5 Present Address: Barnstable County Department of Health and Environment, Sandwich, MA USA; 6 Present Address: Multiphase Chemistry Department, Max Planck Institute for Chemistry, Mainz, Germany

**Keywords:** Water microbiology, Environmental sciences

## Abstract

Hydrothermal sediments contain large numbers of uncultured heterotrophic microbial lineages. Here, we amended Guaymas Basin sediments with proteins, polysaccharides, nucleic acids or lipids under different redox conditions and cultivated heterotrophic thermophiles with the genomic potential for macromolecule degradation. We reconstructed 20 metagenome-assembled genomes (MAGs) of uncultured lineages affiliating with known archaeal and bacterial phyla, including endospore-forming *Bacilli* and candidate phylum *Marinisomatota*. One *Marinisomatota* MAG had 35 different glycoside hydrolases often in multiple copies, seven extracellular CAZymes, six polysaccharide lyases, and multiple sugar transporters. This population has the potential to degrade a broad spectrum of polysaccharides including chitin, cellulose, pectin, alginate, chondroitin, and carrageenan. We also describe thermophiles affiliating with the genera *Thermosyntropha*, *Thermovirga*, and *Kosmotoga* with the capability to make a living on nucleic acids, lipids, or multiple macromolecule classes, respectively. Several populations seemed to lack extracellular enzyme machinery and thus likely scavenged oligo- or monomers (e.g., MAGs affiliating with *Archaeoglobus*) or metabolic products like hydrogen (e.g., MAGs affiliating with *Thermodesulfobacterium* or *Desulforudaceae*). The growth of methanogens or the production of methane was not observed in any condition, indicating that the tested macromolecules are not degraded into substrates for methanogenesis in hydrothermal sediments. We provide new insights into the niches, and genomes of microorganisms that actively degrade abundant necromass macromolecules under oxic, sulfate-reducing, and fermentative thermophilic conditions. These findings improve our understanding of the carbon flow across trophic levels and indicate how primary produced biomass sustains complex and productive ecosystems.

## Introduction

Hydrothermally influenced marine sediments are characterized by fluctuating temperatures, and inorganic and organic carbon substrates that can support a diverse range of microbial metabolic activities [1–3]. The focus of research at hydrothermal systems often lies on the microbial transformation of inorganic compounds and hydrocarbons that originate in hydrothermal fluids and in the subsurface [4, 5]. However, a substantial proportion of the diversity and community function at hydrothermal systems is attributed to heterotrophic microorganisms [6, 7]. This heterotrophic community comprises many uncultured and understudied lineages which likely meet their carbon and energy demands using biological macromolecules such as proteins [8], nucleic acids [9], lipids [10], and polysaccharides [11]. Whether supplied as detritus from the overlying water column [12], as necromass from cell lysis, or as exudates, biological macromolecules are considered the most abundant class of organic compounds available for catabolism in marine sediments [13]. Identifying the microorganisms and enzymes involved in the degradation of macromolecules in hydrothermal sediments is thus relevant for our understanding of biogeochemical cycling and ecosystem functioning in deep-sea environments.

Hydrothermally influenced environments have been shown to host archaeal and bacterial heterotrophs, including *Thermotogales*, *Thermococcales*, and *Archaeoglobales* [14] that are able to grow on biological macromolecules. The use of macromolecules for growth requires a wide range of enzymes involved in extracellular cleavage, transmembrane import, and intracellular degradation. Generally, these polymeric substrates first need to be hydrolyzed by enzymes that are attached to cells or are released to the environment [15]. Subsequently, membrane receptors transport the depolymerized oligo- or monomers into the cell [16], which then enter catabolic pathways. The substrates produced by carbohydrate-active enzymes (CAZymes) and peptidases can be metabolized directly by glycolysis and subsequent oxidation via respiration or fermentation, while nucleosides and fatty acids require further hydrolysis and oxidation before entering the central carbon metabolic pathways [17].

**Fig. 1:**
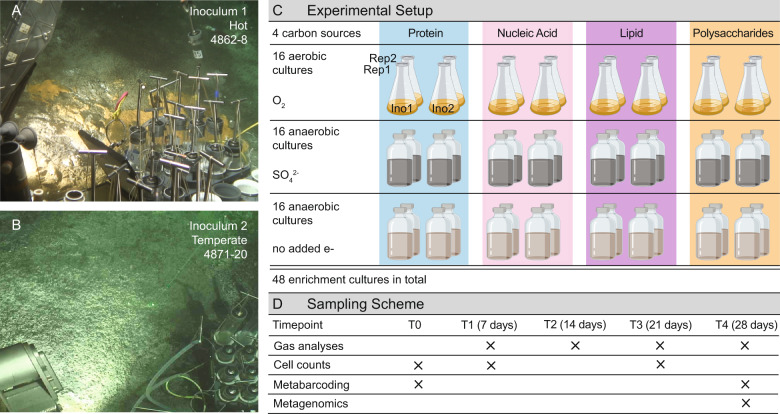
Overview of sampling sites, experimental design and analyses. **A** Core 4862-8 was recovered from hot hydrothermal sediments covered by an orange Beggiatoa mat and characterized by a steep thermal gradient that reached up to 146 °C in 30 cm below seafloor (cmbsf). **B** Core 4871-20 was recovered from temperate sediments characterized by a light-gray patch of sulfur deposits and infaunal worms and a moderate thermal gradient that reached ca. 14 °C in 50 cmbsf. **C** Enrichment cultures from slurries of cores 4862-8 and 4871-20 were supplemented with four types of carbon sources and incubated under oxic, sulfate-amended and fermentative conditions. The resulting 24 cultures were duplicated, yielding 48 enrichments. **D** Timeline of data collection during the period of incubation.

Here we discovered and characterized heterotrophic thermophiles capable of degrading biological macromolecules in hydrothermal sediments. We targeted key metabolisms through amending organic-rich marine sediments from Guaymas Basin, Gulf of California, Mexico, with either proteins, polysaccharides, nucleic acids or lipids as sole added carbon and energy source (Fig. 1A, B). We investigated whether these macromolecule-degrading communities comprise uncultured microbial lineages that have been detected previously in metagenomic surveys of hydrothermal seafloor habitats, providing insights into so far overlooked ecological niches and the vast diversity of heterotrophs in these hydrothermal sediments.

## Methods

### Enrichment cultures

Sediments were collected from two Alvin dives (4862 and 4871) during R/V *Atlantis* research cruise AT37-06 (December 2016) in the Guaymas Basin, Gulf of California, Mexico. Core 4862-8 site was collected from a hydrothermal hot spot near the Mat Mound area (27˚00.43′/111˚24.56′) in the southern Guaymas axial valley [18], and was characterized by an orange *Beggiatoa* mat and a very steep thermal gradient that reached up to 146 °C in 30 cm below seafloor (cmbsf) (Fig. 1A) [19]. Core 4871-20 was collected from a temperate site, termed “Site 2” (27˚02.77′/111˚23.09′) in the “Northern Towers” area, ca. 3 miles to the northeast of the frequently sampled hydrothermal locations [18]; the sediment was characterized by a light-gray patch of sulfur deposits and infaunal worms and a moderate thermal gradient that reached ca. 14 °C in 50 cmbsf (Fig. 1B) [19]. Both cores were strongly sulfidic and gas-rich, as observed immediately after shipboard recovery; oxygen penetrates Guaymas Basin hydrothermal sediments only by 1–2 mm, if at all [18]. Sediment (~150–200 ml) including porewater was sampled from three horizons (0–2, 3–4 and 5–10 cm) and stored without headspace in wide-mouth glass bottles at 4 °C (for details see Supplementary Information). Determination of optimal sample volume for inoculation is provided (see Supplementary Information). Slurries (1:10 sediment:anoxic artificial seawater) of 4862-8 (“hot”) and 4871-20 (“temperate”) sediments served as inocula for enrichment cultures incubated with either proteins, polysaccharides, nucleic acids, or lipids as carbon and energy source, and either oxygen (from air) or sulfate as an electron acceptor, or without an added electron acceptor (fermentative condition), in duplicates (*n* = 48). 50 ml anoxic cultures were grown in 100 ml serum bottles under argon headspace and 50 ml oxic cultures were grown in 250 ml baffled flasks in a shaking incubator (Thermo MaxQ6000). We used basal saltwater medium with sulfate for sulfate-reducing and oxic cultures and basal saltwater medium lacking sulfate (which was replaced by equimolar chloride) for fermentative cultures [20]. Exact concentrations of the media constituents are provided (Table S1). All enrichment cultures were incubated at 60 °C for 4 weeks.

### Choice of carbon sources

Microbial cell lysate contains macromolecular organic matter, including proteins, lipids, nucleic acids, and polysaccharides (e.g., [21]). To test which microorganisms have specialized in the degradation of each of these compound classes, we chose analogues, i.e., model substrates, of defined origin whose structural key linkages (peptide, phosphodiester, and lipid glycerol ester bonds) are targeted consistently by the hydrolytic enzymes for each substrate class [22]. We used bovine serum albumin (BSA) as an analogue for water-soluble protein, because BSA is in the same size range (~600 amino acids, ~60 kDa) as many soluble microbial proteins (e.g., [23, 24]). We chose bacterial DNA of typical gram-negative *Gammaproteobacteria* (genus *Salmonella*), because we can produce it in sufficient quantity and high purity. The phosphatidylcholine lipid that we chose is derived from soy but is widespread in the domain Bacteria as well [25]. Although microbial cells harbor many more different polysaccharides, we included seven polysaccharides (alginate, amylopectin (starch), carrageenan, chitosan, chondroitin, dextran, and xanthan) that represent a variety of monomeric building blocks and glycosyl bonds found in microbial cells and marine algae [26–30].

### Gas measurements

Hydrogen and methane concentrations of the anoxic enrichments were measured weekly using an SRI 310C gas chromatograph with thermal conductivity detector and molecular sieve column (see also Supplementary Information). After each measurement the headspace was purged with Argon to exclude product inhibition by the produced hydrogen. Thus, an increase in hydrogen from one week to another does not only represent an increase in the amount of hydrogen, but also in the rate of its production.

### Cell counts

Cells were enumerated by direct microscopic counts from the cultures during the first and third week of incubation. In brief, the paraformaldehyde fixed cells were sonicated, filtered onto a polycarbonate filter (0.2 µm pore size), stained with DAPI, embedded in Citifluor:Vectashield (4:1) and counted using 20 grids (100 × 100 µm) per sample (see also Supplementary Information).

### Statistical analyses

To determine differential activity (*H*_2_ concentrations and microbial cell numbers) among culture conditions, mixed-effects repeated measures analysis of variance (ANOVA) was employed using the *lme* function within the *nlme* package [31]. When significant effects were found, multiple comparisons between culture conditions were performed using the *glht* function within the *multcomp* package [32].

### DNA extraction

Total nucleic acids were extracted from all 48 enrichments with a modified protocol based on Zhou et al. [33]. In brief, we treated 5 ml of sample using chemical (extraction buffer without CTAB), physical (3× freeze thaw) and enzymatic steps (overnight at 37 °C in lysozyme solution) for extraction of the DNA. Extraction blanks were included to assess potential laboratory contamination during extraction. DNA concentrations were measured fluorometrically using a Qubit 2.0 fluorometer (Thermo Fisher Scientific, Canada).

### 16S rRNA gene amplicon sequencing and processing

The 16S rRNA gene V4-V5 variable regions were amplified using the bacterial primers 518F and 926R [34] and the archaeal primers 517F and 958R [35] (for details see Supplementary Information). Amplicons were sequenced using Illumina’s v3 600-cycle (paired-end) reagent kit on a MiSeq (Illumina Inc., San Diego, CA, USA). Reads were demultiplexed based on the combination of index (CASAVA 1.8) and barcode (custom python scripts). Raw sequences were processed and amplicon sequence variants (ASVs) were generated following the DADA2 [36] Pipeline Tutorial v1 (https://benjjneb.github.io/dada2/tutorial.html), for details see Supplementary Information.

### 16S rRNA-based community analyses

Community composition, diversity indices, and dissimilarity matrices were obtained using ASV relative abundances. Data analysis was performed using VisuaR (https://github.com/EmilRuff/VisuaR), a workflow based on custom scripts and R packages, including *vegan* v2.5-6 [37] and *ggplot2* v3.3.2 [38] (for details see Supplementary Information).

### Metagenomic sequencing, assembly, binning and analyses

Total DNA from 11 samples was used to prepare libraries with an Ovation Ultralow V2 DNA-Seq Kit (TECAN Group Ltd., Mannedorf, Switzerland). Sequencing was performed on an Illumina NextSeq 550 using the paired-end 2 × 150 bp run-type mode. Quality control was performed using PRINSEQ [39] (for details see Supplementary Information). Reads were co-assembled using SPAdes with the --meta option [40] and binned using MetaWRAP processing modules with initial extraction using MaxBin2, metaBAT2, and CONCOCT, bin refinement, and reassembly [41] (for details see Supplementary Information). Assembled genomes with more than 90% completeness, less than 3% contamination, as assessed using CheckM [42], and less than 100 contigs were further analyzed (Table 1). Taxonomy and closest phylogenetic relatives were assigned using GTDB-tk (v1.3.0) [43], average nucleotide identity (ANI), average amino acid identity (AAI) (Supplementary Data 1), and single-copy genes. A maximum likelihood phylogenetic tree of genomes was generated using PATRIC RaxML service [44] and visualized using the Interactive Tree of Life webtool.

**Table 1:** Summary statistics for 3 archaeal and 17 bacterial metagenome-assembled genomes (MAGs) enriched in thermophilic cultures using Guaymas Basin sediments.

GTDB taxonomy GB_ID	Genome size (Mb)	GC (%)	CheckM^a^ completeness	CheckM contamination	Number of scaffolds	OrthoANIu value (%)^b^	AAI value (%)^c^	Quality^d^	Phylum
*Thermococcus* sp. GB_027	2.05	40	99.5	0.5	25	89	91	Medium	Euryarchaeota
*Archaeoglobus* sp. GB_100	2.04	46	98.04	0	92	80	85	High	Halobacteriota
*Archaeoglobus* sp. GB_049	2.55	43	100	1.31	83	71	66	Medium	Halobacteriota
UBA2242 GB_043	2.80	34	97.8	1.1	50	64	50	Medium	Marinisomatota
UBA2242 GB_103	2.66	40	97.8	0	38	64	51	Medium	Marinisomatota
*Thermodesulfobacterium* sp. GB_111	1.67	33	98.75	0.21	43	80	79	Medium	Desulfobacterota
*Kosmotoga* sp. GB_055	2.15	41	98.28	1.72	28	79	84	Medium	Thermotogota
*Thermosipho* sp. GB_064	2.12	32	98.09	0	88	77	77	Medium	Thermotogota
*Thermosipho* sp. GB_058	1.79	30	100	0	33	77	77	Medium	Thermotogota
*Thermovirga* sp. GB_013	1.82	44	100	0	47	75	81	Medium	Synergistota
*Bacillus* sp. GB_116	3.11	39	98.34	0.55	42	68	63	Medium	Firmicutes
*Melghiribacillus* sp. GB_076	3.24	41	98	1.33	25	77	80	Medium	Firmicutes
*Brevibacillaceae* GB_007	3.41	47	90.55	2.32	36	67	53	Medium	Firmicutes
*Brevibacillus* sp. GB_061	3.60	54	98.39	0.54	31	72	67	Medium	Firmicutes
*Calderihabitantaceae* GB_131	2.73	47	98.98	0.65	32	68	53	Medium	Firmicutes
*Thermosyntropha* sp. GB_081	2.11	39	99.23	0	15	74	76	Medium	Firmicutes
ZCTH02-B6 sp. GB_124	2.59	65	97.06	0.98	77	72	70	Medium	Firmicutes
UBA5301 sp. GB_067	1.91	53	97.46	1.69	86	67	67	Medium	Firmicutes
*Desulforudaceae* GB_121	2.31	50	99.91	1.49	82	67	60	Medium	Firmicutes
*Desulfohalotomaculum* sp. GB_002	2.31	43	99.37	0.63	77	70	66	Medium	Firmicutes

^a^CheckM values were calculated based on [42].

^b^Average nucleotide identity (ANI) values to the closest isolated or candidate relative [107]. Conservative phylogenetic thresholds: same species >90%, same genus >60% [108].

^c^Average amino acid identity (AAI) values to the closest isolated or candidate relative. Conservative phylogenetic thresholds: same species >90%, same genus >65%, same family >45% [109].

^d^Quality was assessed using completeness and contamination [54].

Genomes were annotated using Distilled and Refined Annotation of Metabolism (DRAM) [45], with default parameters. DRAM searches amino acid sequences against multiple databases such as KEGG, MEROPS and dbCAN/CAZYmes, and provides all database hits in a single output file. We searched for key genes in the raw (Supplementary Data 2) and metabolism summary files (Supplementary Data 3) provided by DRAM.py annotate and DRAM.py distill, respectively. Localization analyses for genes of interest were done using PSORTb [46]. We classified the metagenome-assembled genomes (MAGs) as potential degraders of the carbon substrates based on their presence in enrichment cultures amended with the particular macromolecule and the presence of enzymes involved in extracellular cleavage, transmembrane import, and intracellular degradation of the particular macromolecule in their genome (Supplementary Information).

### Abundance of MAGs

The abundances of MAGs across enrichment cultures and in environmental metagenomes were estimated using the metaWRAP Quant bins module [41]. Quant bins uses Salmon [47] to align reads from each sample to the assembly contigs producing coverage values for each contig. These coverage values (CPM) are standardized by library size (for every 1,000,000 metagenomic reads) and by contig length, similar to transcripts per million (TPM) in RNAseq analysis. Then, Quant bins estimates the abundance of MAGs in each sample by computing a length-weighted average of the MAG’s contigs CPMs. We analyzed published metagenomes of hydrothermal ecosystems in Guaymas Basin [7, 48, 49] (16 datasets), of the Mid-Atlantic Ridge (4 datasets) [50], of Okinawa Trough (4 datasets) [51] and Brothers submarine volcano (6 datasets) [52], and of hadal sediments from the Yap Trench (3 datasets) [53] (Supplementary Data 4).

## Results

### Experimental design summary

Hot and temperate hydrothermal sediments from the southern spreading center of Guaymas Basin (Fig. 1A, B), were amended with either proteins, nucleic acids, lipids or polysaccharides as sole added carbon and energy source at 60 °C under oxic, sulfate-reducing, or fermentative (no added electron acceptor) conditions (Fig. 1C). Enriched bacterial and archaeal lineages and their catabolic potential were analyzed by 16S rRNA gene and metagenomic analyses (Fig. 1D), methane and hydrogen production was measured using gas chromatography and growth was estimated using cell counts.

### Hydrogen, not methane, is a product of anoxic macromolecule degradation

**Fig. 2:**
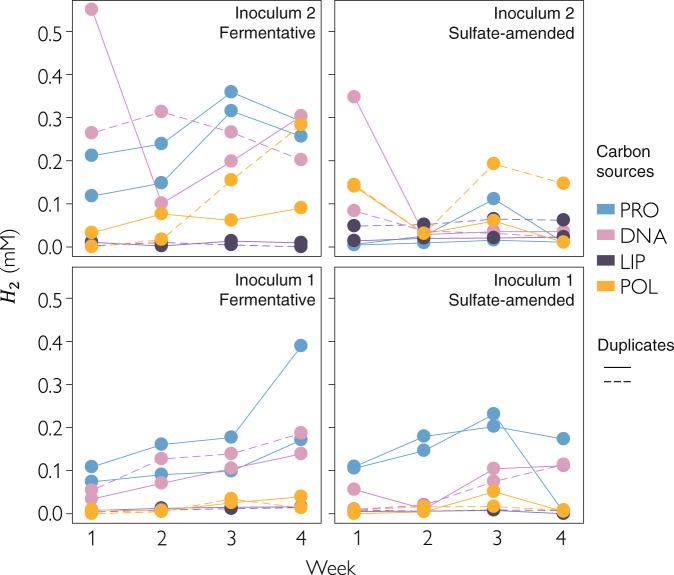
Hydrogen production in thermophilic enrichment cultures. Hydrogen was measured weekly in sulfate-reducing and fermentative anoxic cultures that were supplemented with proteins (PRO), nucleic acids (DNA), lipids (LIP), or polysaccharides (POL) having a temperate or a hot sediment as inoculum. The headspace was exchanged every week, hence a straight line represents constant production, and increasing line means increasing production of hydrogen per time.

To monitor the enrichment of anaerobic, heterotrophic thermophiles, we followed the production of methane and molecular hydrogen, key products of methanogenesis and fermentation respectively, in the headspace of anoxic cultures weekly (1–4 weeks) (Fig. 2). We did not detect methane in any of the enrichments, despite a detection limit of 200 ppm (8 µM methane). In addition, we did not find evidence of methanogens in the 16S rRNA gene and metagenomic sequencing datasets. Thus, enrichments under anoxic conditions that were expected to support methanogenesis apparently did not contain a syntrophic network including methanogens. However, we observed an increase in the concentration of *H*_2_ in the headspace with time, indicating a successful enrichment of fermentative microbes (Fig. 2). Fermentative enrichments had significantly more hydrogen than the sulfate-containing cultures (*p* < 0.0001, Table S2), while *H*_2_ concentrations were highest in cultures supplemented with proteins or DNA and lowest in cultures supplemented with lipids (Fig. 2, Table S3).

The rate of *H*_2_ production was associated with microbial growth over time, as the production of *H*_2_ and the cell counts positively correlated (*R*^2^: 0.35, *p* = 0.048). At week 3, the microbial cell numbers were highest in cultures supplemented with polysaccharides or proteins and lowest in cultures supplemented with lipids (Fig. S1a), indicating that proteins and polysaccharides were turned over faster or were more labile than DNA and lipids (Fig. S1b). Although we did not constrain how much of the added carbon was consumed, we can estimate how much carbon was used to produce new cells (Supplementary Information, Supplementary Data 5). Based on our estimates using the increase in cell numbers, about 6.6% of the added polysaccharide-carbon was used for the production of cells. For proteins (4.5%), DNA (4.2%), and lipids (2.2%), this percentage was lower (Supplementary Data 5).

### Macromolecule type and redox conditions selectively enriched for distinct communities

**Fig. 3:**
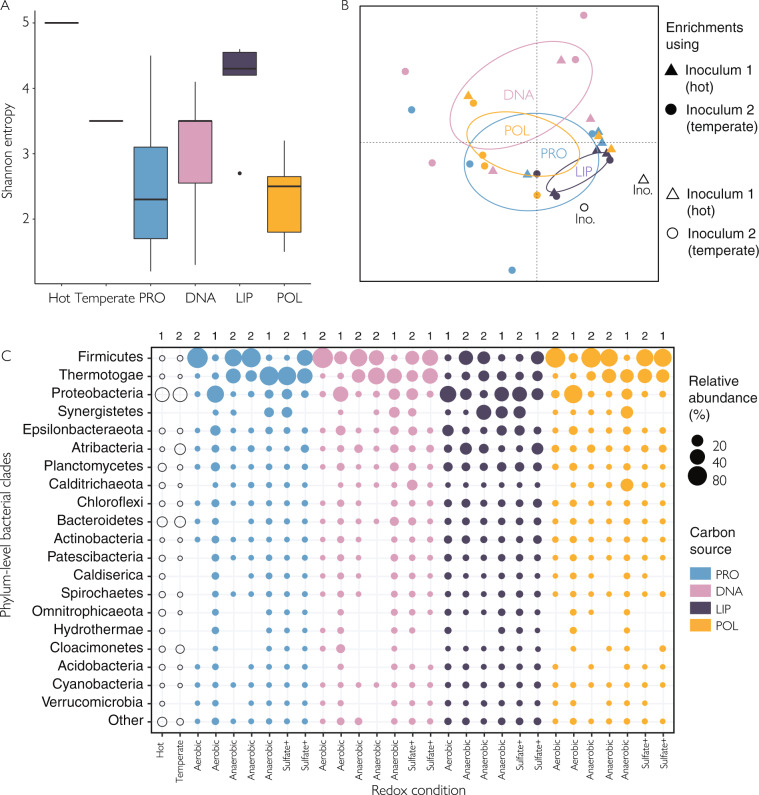
Bacterial community structure of Guaymas Basin thermophilic enrichment cultures. **A** Shannon’s entropy, **B** non-metric multidimensional scaling (NMDS) analyses (95% confidence ellipse) and **C** relative sequence abundance of the most abundant bacterial clades in the temperate and hot sediment used as inocula and in enrichment cultures supplemented with proteins (PRO), nucleic acids (DNA), lipids (LIP) or polysaccharides (POL) under oxic, sulfate-reducing or fermentative (no added electron acceptor) conditions after four weeks of incubation. The used inoculum is indicated at the top of the graph. Relative sequence abundances are based on 16S rRNA V4/V5 gene amplicons.

The differences in *H*_2_ production and cell counts coincided with differences in microbial community structure. Protein-amended cultures (with high *H*_2_ production) displayed a decreased bacterial diversity, indicating selective enrichment of specific taxa, while lipid-amended cultures (low *H*_2_ production) retained much of the original diversity (Fig. 3A, B). In the hot and temperate sediment, *Proteobacteria* and *Bacteroidetes* accounted for ~40 and ~20% of the 16S rRNA gene amplicons, respectively. After 4 weeks of incubation, the relative sequence abundance of *Proteobacteria* and *Bacteroidetes* decreased to an average of 16% and 1%, respectively (Fig. 3C). *Firmicutes* increased from 1% in both inocula, to an average of 41% across all enrichment cultures (Fig. 3C). The PerMANOVA results based on genus-level bacterial relative sequence abundance detected significant differences in community structure between redox conditions (*p* = 0.008, *R*^2^ = 0.15) as well as between carbon sources (*p* = 0.010, *R*^2^ = 0.20). However, significant post-hoc comparisons were only found between oxic and anoxic conditions (Fig. S2, *P* values are reported in Table S4). Lineages found in more than one culture condition, low number of samples, and variation between replicates potentially limited our statistical power (Fig. S3). Differences in archaeal community composition also reflected a selective enrichment of microbes affiliated with specific taxa. In the hot sediment inoculum, anaerobic methane-oxidizing archaea of the ANME-1 lineage accounted for 75% of the archaeal 16S rRNA gene amplicons. After 4 weeks of incubation, the relative sequence abundance of ANME-1 decreased in most of the enrichment cultures, while *Thermococcus* increased from 3% in the hot sediment to an average of 51% across enrichment cultures (Fig. S4).

### Uncharacterized bacterial lineages are involved in the degradation of biological macromolecules

**Fig. 4:**
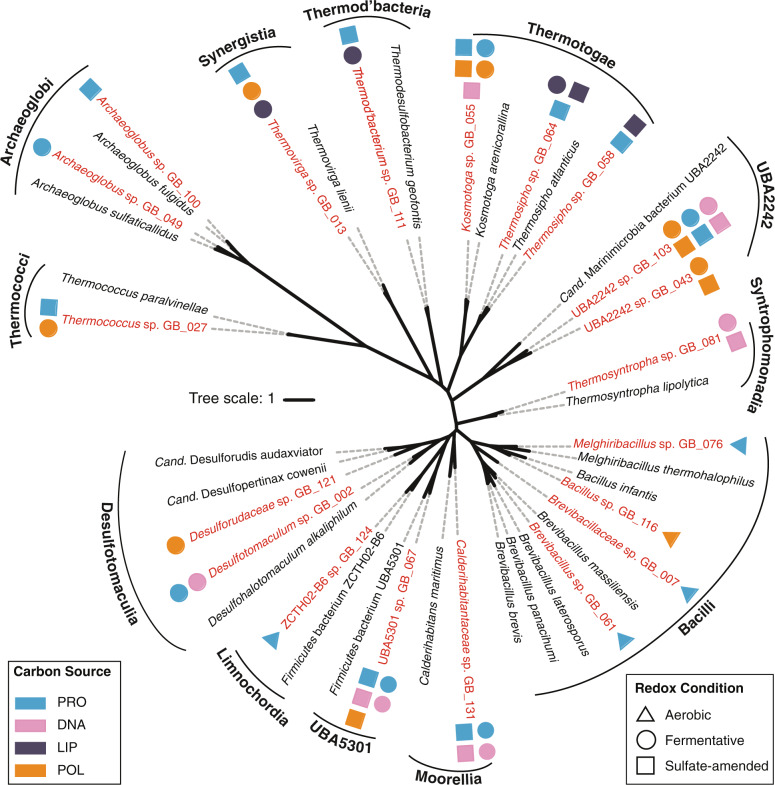
Maximum likelihood phylogenetic tree of MAGs reconstructed from Guaymas Basin thermophilic enrichment cultures (red) and closest relatives (black) based on single-copy genes. Culture conditions enriching MAGs are shown. Frequently, different enrichment conditions (carbon sources and redox conditions) selected for the same MAG, as determined by mapping the sequences of each MAG to the metagenomes of each cultivation regime.

**Fig. 5:**
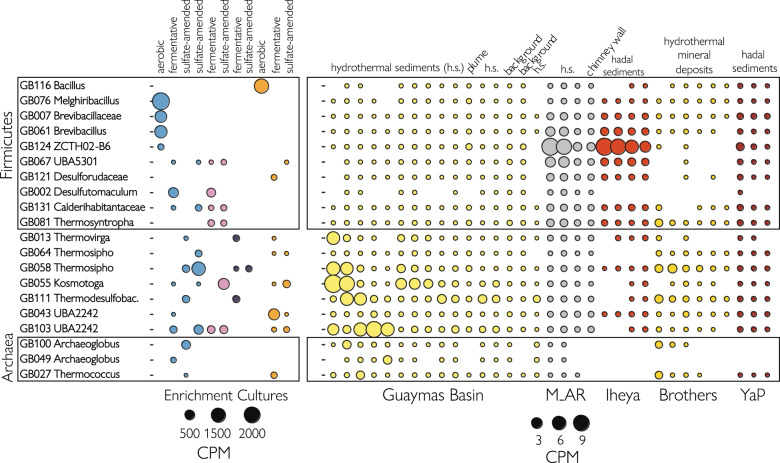
Abundance estimates of MAGs across enrichment cultures and environmental metagenomes. (Left) Guaymas Basin enrichment cultures supplemented with proteins (blue), nucleic acids (pink), lipids (purple) or polysaccharides (orange) under oxic, sulfate-reducing or fermentative conditions. (Right) Environmental metagenomes from Guaymas Basin, Mid-Atlantic Ridge, Iheya Ridge and Yap Trench. The abundance estimates are expressed as contigs per million reads (CPM) calculated with metaWRAP’s Quant bins module. Quant bins uses Salmon to align reads from each sample to the bins producing coverage values for each contig in the same manner as transcripts per million (TPM). The abundance of each MAG in each sample is calculated by taking the length-weighted average of the MAG’s contig coverage.

Metagenomic sequencing, assembly and binning of DNA extracted from 11 cultures revealed 3 archaeal and 17 bacterial medium-to-high-quality MAGs [54]. These MAGs were assigned to seven phyla, with a majority affiliating with *Firmicutes* (Table 1). The taxonomic analyses from GTDB-tk, ANI, AAI, and single-copy genes confirmed that all MAGs represented populations of new species (Supplementary Data 1). Four MAGs were affiliated with the uncultivated lineages UBA2242, UBA5301, and ZCTH02-B6 (Fig. 4). The class-level UBA2242 lineage falls within the candidate phylum *Marinisomatota*/“*Candidatus* Marinimicrobia” (previously Marine Group A or candidate phylum SAR406) and the class-level lineage UBA5301 falls within the phylum *Firmicutes* [55]. The ZCTH02-B6 lineage is an uncultured family-level lineage within the phylum *Firmicutes* and affiliated with the class *Limnochordia* [56]. Three MAGs that were unclassified at the genus-level were assigned to the families *Brevibacillaceae*, *Calderihabitantaceae*, and *Desulforudaceae* (Supplementary Data 1) within the phylum *Firmicutes* (Fig. 4). The 13 remaining MAGs could be assigned to known genera (Supplementary Data 1). All *Firmicutes* MAGs encoded genes for sporulation (Supplementary Data 6). MAG abundance estimates in each set of culture conditions revealed that several MAGs were in high abundance only under one enrichment regime, while others were found in several conditions (Fig. 5), suggesting specialist and generalist lifestyles. Moreover, the abundance estimates revealed that the MAGs represent lineages with widespread occurrence in other hydrothermal and sedimentary environments. Particularly, high coverages of UBA2242, *Thermotogae*, *Thermodesulfobacteria*, and *Synergistia* populations were found in environmental samples of Guaymas Basin hydrothermal sediments (Fig. 5). *Brevibacillus*, ZCTH02-B6, and UBA5301 lineages were abundant in hydrothermal sediments from the Mid-Atlantic Ridge and Okinawa Trough (Iheya Ridge).

### Thermophilic protein degrading populations

**Fig. 6:**
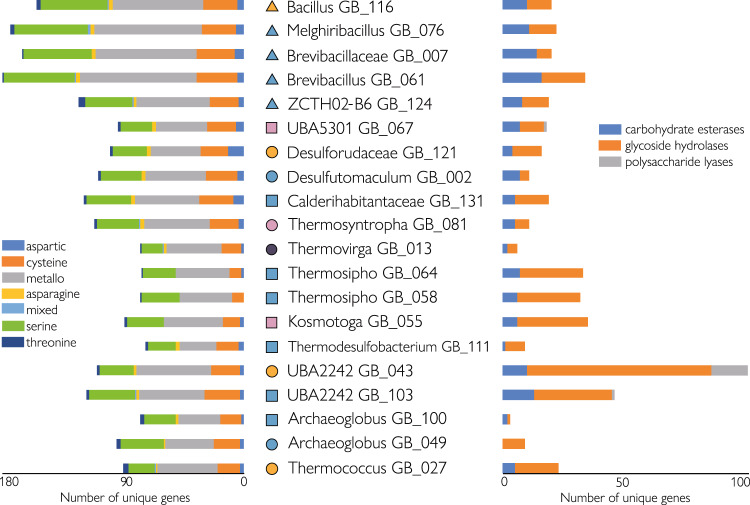
Number of proteolytic enzymes by gene family (left) and carbohydrate-active enzymes (CAZymes) by enzyme classes (right) found in the MAGs. Main culture conditions enriching MAGs are shown: proteins (blue), nucleic acids (pink), lipids (purple) or polysaccharides (orange) under oxic (triangle), sulfate-reducing (square) or fermentative (circle) conditions. A complete list of the annotations is reported in Supplementary Data 2 (peptidases) and 3 (CAZymes).

**Fig. 7:**
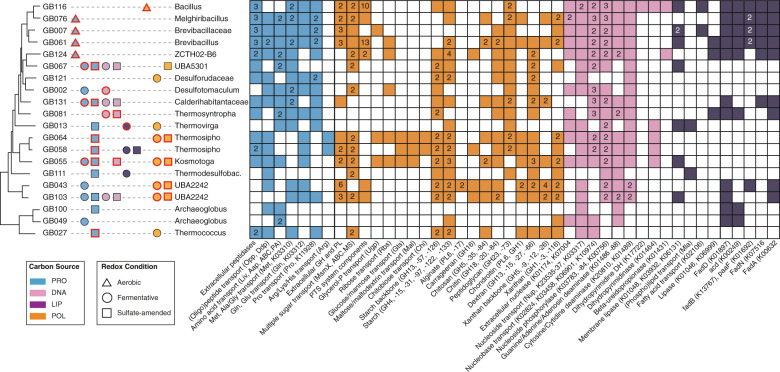
Presence of key genes encoding protein (blue), polysaccharide (orange), nucleic acid (pink), and lipid (purple) degradation and transport in Guaymas Basin thermophilic enrichment cultures MAGs. Culture conditions enriching MAGs are shown: protein (blue), nucleic acid (pink), lipid (purple), polysaccharide (orange), oxic (triangle), sulfate-reducing (square), or fermentative (circle). Culture conditions for which a given MAG encoded the necessary extracellular enzymes are shown with symbols having a red outline. Different capabilities belonging to the same category are given as numbers, e.g., if a MAG has three different extracellular peptidases the blue square in column 1 will show the number 3, if the MAG has only one extracellular peptidase only the blue square is shown. A complete list of the annotations is reported in Supplementary Data 2, 3.

**Fig. 8:**
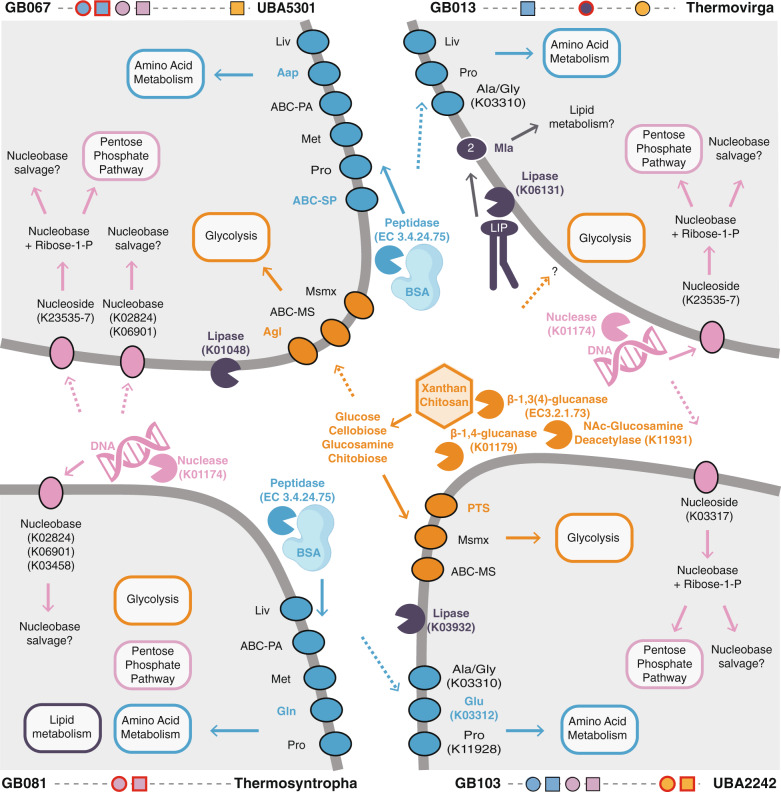
Conceptual overview of metabolic niches and potential metabolic interactions of four selected population genomes (MAGs). Shown are each MAGs major extracellular enzymes and transporters necessary to make a living on protein (blue), nucleic acid (pink), lipid (purple), and polysaccharides (orange). Extracellular enzymes are depicted close to the MAG in which they occur. Arrows represent processes that each MAG is capable of, while dotted arrows show potential flow of monomers/oligomers provided by other MAGs. As an example, GB067 has extracellular peptidases, transporters, and enzymes to grow on proteins. Despite lacking the extracellular nucleases and CAZymes GB067 was also abundant in the nucleic acid and polysaccharide enrichments, likely because it contained the necessary transporters and catabolic pathways to scavenge monomers. Transporters that occur in only one of the population genomes are shown in colored font, e.g. Aap, ABC-SP and Agl were only present in MAG GB067.

Protein degradation under oxic conditions was apparently carried out by *Bacilli* and *Limnochordia* (ZCTH02-B6), whose MAGs were enriched in the oxic protein-fed cultures (Fig. 5) and contained the highest number of genes for extracellular and cellular peptidases (Figs. 6, 7, Supplementary Data 7), as well as peptide and amino acid transporters (Fig. 7). The communities that anaerobically degraded proteins were more diverse. These cultures included populations that had extracellular peptidases and the means for peptide uptake (UBA5301, *Calderihabitantaceae*, *Thermotogae*, *Thermococcus*), as well as populations that were only able to uptake peptides or amino acids (*Desulfotomaculum, Thermovirga, Thermodesulfobacterium, UBA2242, Archaeoglobus*) (Figs. 7 and 8).

### Thermophilic polysaccharide degrading populations

Polysaccharide degradation under oxic conditions was largely carried out by a population affiliating with the genus *Bacillus* (Table S5). Like other *Bacilli*, this population did not occur in other conditions although it had the capability to process all provided substrates. Analogous to anaerobic protein degradation, the anaerobic polysaccharide degrading communities contained populations with all necessary enzymes (*Thermosipho*, *Kosmotoga*, and UBA2242), populations that can uptake oligo- /monomers (*Thermococcus*, UBA5301), and populations that apparently lacked the enzymes for extracellular cleavage and uptake (*Desulforudaceae, Thermovirga*) (Figs. 7 and 8). *Thermotogae* populations had very broad polysaccharide-degrading capabilities including several extracellular CAZymes (Supplementary Data 7), numerous sugar transport systems, as well as CAZymes belonging to glycoside hydrolase families which include enzymes to process almost all the polysaccharides we provided (Fig. 7). *Kosmotoga* MAG GB055, for example, encoded two extracellular CAZymes (beta-N-acetylglucosaminidase and 1,4-beta-cellobiosidase) that may be involved in the degradation of chitosan (chitin) and xanthan. This population also had the necessary transporters and CAZymes to make a living on these substrates (Fig. 7). The capabilities of the *Thermotogae* were only rivaled by the two *Marinisomatota* MAGs GB43 (and GB103). These populations were apparently able to degrade a large number of different polysaccharides and had 6 (3) extracellular CAZymes (e.g., chitin deacetylase, endo-beta-1,3(4)-glucanase and 1,4-beta-cellobiosidase) likely involved in the degradation of chitosan and xanthan (Figs. 8 and S5). The MAGs also encoded 6 (1) polysaccharide lyases and CAZymes belonging to 35 (21) different glycoside hydrolase families, which comprise enzymes for the degradation of all seven provided polysaccharides including alginate, chondroitin, and carrageenan. The *Marinisomatota* MAGs lacked most of the common sugar transporters that were found in the other populations, yet contained many genes annotated as TonB proteins (K03832) and TonB-dependent receptors.

### Thermophilic nucleic acid or lipid degrading populations

Although the majority of the MAGs enriched in this study were derived from cultures supplemented with protein and polysaccharides, several bacterial lineages were reconstructed from cultures supplemented with nucleic acids or lipids. MAGs affiliating with the genera *Thermosyntropha*, *Kosmotoga*, *Desulfohalotomaculum*, the family *Calderihabitantaceae*, and the uncultured classes UBA5301 and UBA2242 were found in cultures supplemented with DNA (Fig. 5). *Thermosyntropha*, *Kosmotoga*, and *Desulfohalotomaculum* MAGs featured an extracellular nuclease (EC 3.1.31.1, Fig. 7, Supplementary Data 7). *Kosmotoga*, *Desulfohalotomaculum*, and UBA5301 also contained nucleoside transporters in addition to an extracellular nuclease (Fig. 8). Known genes for purine and pyrimidine degradation were found in UBA2242 and *Kosmotoga* (Fig. 7). *Thermovirga*, *Thermosipho*, and *Thermodesulfobacterium* MAGs were derived from cultures supplemented with lipids. *Thermovirga* featured a membrane phospholipase, phospholipid transporters and long-chain acyl-CoA synthetase (Fig. 8), while *Thermodesulfobacterium* encoded phospholipid transporters (Fig. 7).

### Central carbon and energy metabolisms

**Fig. 9:**
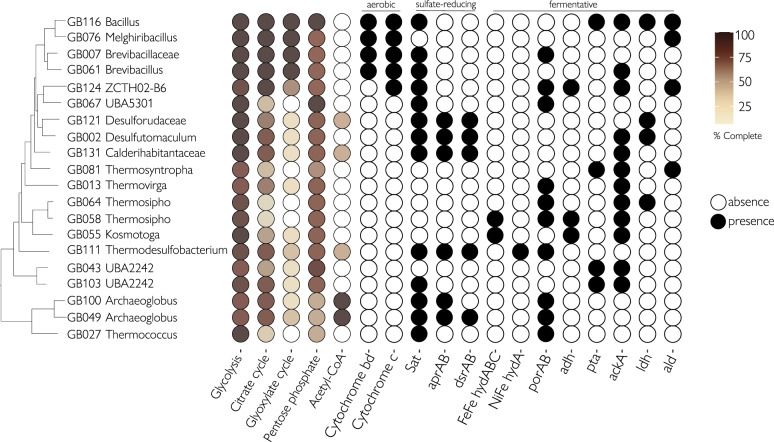
(Left) Central carbon metabolism pathways and (Right) presence of gene sequences involved in aerobic, anaerobic sulfate reduction and fermentation in the metagenome-assembled genomes. A complete list of the annotations is reported in Supplementary Data 2.

The use of biopolymers and lipids as an energy source was also reflected by the presence of genes encoding pathways for the degradation of monomeric substrates and smaller molecules produced by the activity of peptidases, CAZymes, lipases, and nucleases in the MAGs. All *Bacilli* MAGs that were enriched in oxic cultures contained a complete glycolysis pathway, citrate (TCA/Krebs cycle) and glyoxylate cycles, and cytochrome c oxidases (Fig. 9). Pathways for sulfate reduction and fermentation were detected in MAGs under these respective anoxic conditions. *Desulfohalotomaculum*, *Desulforudaceae*, *Calderihabitantaceae*, *Thermodesulfobacterium*, and *Archaeoglobus* featured genes for dissimilatory sulfate reduction (*sat, aprAB*, and *dsrAB*) (Fig. 9). These populations likely couple the oxidation of macromolecule-derived carbon to the reduction of sulfate. In *Thermotogae* we found FeFe hydrogenases (hydABC) which have been shown to be involved in fermentative hydrogen evolution (Fig. 9). These hydrogenases likely contributed to the increased hydrogen production we observed in the fermentative enrichments. Genes that could support fermentative metabolism, phosphate acetyltranferase (pta) and acetate kinase (ackA), were found in *Thermosyntropha* and UBA2242 MAGs (Fig. 9).

## Discussion

### Uncultured heterotrophs contribute to macromolecule degradation in hydrothermal sediments

We combined cultivation with metagenomics to gain insights into heterotrophic Bacteria and Archaea with the potential for protein, polysaccharide, nucleic acid, and lipid degradation in hydrothermally influenced sediments. Each of the tested cultivation conditions enriched a microbial community that was capable of metabolizing the supplemented carbon sources. In previous Guaymas Basin sequence-based surveys [1, 6, 7, 57–62] and in the sediment cores analyzed here, *Firmicutes* were infrequently detected. Yet, we found *Firmicutes* at high relative sequence abundances in many of the enrichment cultures, and half of the MAGs (10 of 20) were affiliated with uncultured *Firmicutes* lineages. The cultivation conditions apparently caused the germination of bacterial thermospores present in Guaymas Basin sediments. Cultivated *Firmicutes* contained sporulation proteins and were closely related to spore formers [63–66]. Spore-forming lineages were found in oxic (*Bacilli*) and anoxic conditions (*Moorellia* and *Desulfotomaculia*).

The presence of sulfate-reducing *Firmicutes* also indicates a potential role of these lineages in thermophilic sulfate reduction in Guaymas Basin [67]. Previous studies have shown that high temperature incubation of cold sediments activates and supports the growth of endospore-forming bacteria via sulfate reduction and fermentation [68]. Under suitable environmental conditions these spores germinate and may become major contributors to community functions in the warm hydrothermal sediments, which potentially represent a source of thermospores widely distributed in marine sediments [69, 70]. For example, we detected the *Firmicutes* uncultured lineages UBA5301 and ZCTH02-B6 in hydrothermal sediments from Mid-Atlantic and Okinawa Trough. ZCTH02-B6 protein- and UBA5301 protein- and polysaccharide- degradation capabilities suggest that sediments in these environments provide the particular substrates and can sustain heterotrophic communities. Interestingly, the closest relatives of our unclassified *Bacillaceae* MAGs such as *Brevibacillus brevis* are not associated with hot environments [66], expanding the habitat range of this group. Further research should address the question whether these organisms originate from deeper sediment layers and reach the surface via geofluids [69, 71] or whether they are deposited by ocean currents [72].

The enrichment of uncultured lineages such as UBA2242 within the candidate phylum *Marinisomatota* underlines the successful cultivation of uncultured diversity of Guaymas Basin. The candidate phylum *Marinisomatota* is considered a highly diverse group, participating in marine sulfur and nitrogen cycles [73–75]. *Marinisomatota* (formerly SAR406, Marine Group A, *Marinimicrobia*) was reported to comprise lineages that degrade complex carbohydrates in marine oxygen minimum zones [76] featuring numerous glycoside hydrolase families with enzymes involved in the degradation of chitin, cellulose, starch, sulfated galactans among others (Fig. S6). Our finding of two *Marinisomatota* MAGs with similar capabilities suggests that the ability to degrade complex polysaccharides may be more widespread among *Marinisomatota*. Besides Guaymas Basin [7], UBA2242 have been previously detected in cold-water geyser fluids in Utah, USA [77].

The microbial utilization of environmental DNA, either to salvage nucleosides or as a carbon and energy source, was shown to occur in marine sediments [78–81]. At least nine of the enriched thermophiles had the capability to extracellularly cleave DNA, take up the nucleosides, remove the ribose and use it as carbon and energy source via the pentose phosphate pathway (Figs. 7 and 9). GB116 affiliating with *Bacillus* additionally had the enzymes to further catabolize pyrimidines (Fig. 7). However, despite having extracellular nucleases, nucleoside transporters, and nucleoside phosphorylases not all of these populations were enriched in the DNA-amended cultures (Figs. 7 and 9). The widespread metabolic capabilities and the general abundance of DNA in marine sediments [9] suggest that nucleic acid degradation is a common process and ecological niche in hydrothermal sediments.

Lipids have also been shown to be microbially degraded in marine sediments [82, 83] and serve as carbon and energy source for microorganisms [10, 84]. Lipids were apparently the most difficult substrate for the enriched thermophiles promoting the lowest increase in cell numbers and the least community shifts. Surprisingly, the populations that were most abundant in the lipid-amended microcosms seemed to lack most (GB013—*Thermovirga*, GB111—*Thermodesulfobacterium*) or all genes (GB058—*Thermosipho*) that are needed to import and metabolize the lipids or fatty acids (Figs. 7 and 9). These organisms thus may not use lipids after all, but other organic compounds released from the perished cells of the inoculum. Unknown genes, pathways, or processes, e.g., non-classical secretion of extracellular enzymes [85], or novel metabolisms could also explain this gap in knowledge and merit further investigation.

### Niches of thermophilic heterotrophs involved in macromolecule degradation

The findings of our metabolic analyses suggest that Guaymas Basin sediments harbored specialists that were adapted to the utilization of a particular carbon polymer (e.g., *Brevibacillales*) as well as generalists that were able to degrade a wide range of biopolymers under different redox conditions (e.g., *Thermotogae*) (Fig. 8). Most of the cultivated lineages were capable of secreting enzymes, suggesting the ability to hydrolyze the polymers into oligomers extracellularly. The lineages that lacked exoenzymes (e.g., *Archaeoglobus*) contained oligomer transporters instead. These populations potentially have opportunistic trophic interactions with exoenzyme-producing decomposers and feed on the products of extracellular hydrolysis (Fig. 8) carried out by other microbes [86]. We found that protein and polysaccharide degradation is likely carried out by multiple bacterial and archaeal lineages. Consistent with the literature, *Archaeoglobus* species were capable of coupling the oxidation of carbon compounds to the reduction of sulfate [87, 88], *Thermococcus* were thriving on peptides and carbohydrates and likely produced hydrogen [89], and *Thermotogae* and *Marinisomatota* were able to degrade a wide range of polysaccharides [90, 91].

Nucleic acids and lipids were found to be less labile or turned over slower than proteins and polysaccharides, especially the slow growth on lipids in anoxic conditions is consistent with previous findings [10]. *Thermosyntropha* and *Thermovirga* however thrived in cultures supplemented with nucleic acids and lipids, respectively. Although DNA and lipids are energy-rich and abundant in marine sediments [9], the identity and distribution of nucleases and lipases across genomes, organisms and databases is not well characterized, in contrast to the enzymes involved in the breakdown of proteins [92, 93] and polysaccharides [94, 95]. Few studies have identified bacterial extracellular nucleases [96, 97] and lipases [98] associated with catabolic activities. Furthermore, nucleases and lipases can also participate in synthesis and salvage pathways, and are not necessarily involved in catabolism [99, 100]. Interestingly, although the energy yield of all provided substrates is highest using oxygen as electron acceptor [101], we did not find populations that were specialized on the aerobic degradation of lipids and nucleic acids. Overall, despite the environmental pressure of high temperature and minimal media the enriched food webs apparently contained primary degraders, secondary degraders and potentially even hydrogen-oxidizing sulfate reducers revealing insights into heterotrophic food webs and the ecophysiology of uncultured lineages [102].

### Implications of macromolecule degradation for marine carbon cycling

We show that Guaymas Basin hydrothermal sediments host a variety of thermophilic heterotrophs with the potential to hydrolyze the tested cellular macromolecules and transport their oligomers. The oligomers can be further oxidized via glycolysis fed respiration and fermentation. Although we provided conditions that are considered suitable for hydrogenotrophic methanogenesis, methane was not detected in the headspace of any culture and we were not able to enrich hydrogenotrophic, acetoclastic, or methylotrophic methanogens. This indicates that thermophilic macromolecule degradation does not support methanogenesis or syntrophic interactions required to cross-feed methanogens under the tested conditions. Stable carbon isotopic evidence for methanogenesis (δ^13^C for porewater methane near −70‰) in Guaymas Basin sediments was limited to cool sediments, possibly due to overprinting by microbial methane oxidation [1]. Previous anoxic high temperature (50 °C) incubations of Guaymas sediments over 5–9 days have resulted in hydrogen accumulation [59], suggesting that hydrogenotrophic methanogens were not enriched under these conditions either. Hyperthermophilic hydrogenotrophic methanogens have been isolated from Guaymas Basin sediments [103], and the key genes of methanogenesis, methyl coenzyme M reductase (mcrA), including those of hyperthermophilic lineages, are found in Guaymas Basin sediments [104]. Hence, these methanogenic populations may respond to hyperthermophilic enrichments (80 °C). Thermophilic *Methermicoccaceae* were found in Guaymas sediments [59], yet these organisms are obligately methylotrophic and thus may have not found suitable substrate in our enrichments. Hydrogenotrophic and acetoclastic methanogens, on the other hand, may have been too low in abundance in the inoculum, due to being inhibited by methane or outcompeted by sulfate reducers [105]. However, most evidence for the presence of hydrogenotrophic, acetoclastic, and methylotrophic methanogens in Guaymas Basin sediments is based on gene sequencing, which may underrepresent active but infrequently occurring community members [106]. In cold and temperate anoxic sediments, it was shown that methane was produced from e.g., lipid degradation [10, 83]. The finding that methane was not produced during thermophilic degradation of biomass constituents thus merits further investigation and if confirmed has implications for the deep-sea methane cycle. Biomass carbon that was assimilated using subsurface-derived methane at hydrothermal sediments may not be converted back into methane, but into carbon dioxide and organic acids.

## Conclusion

The heterotrophic populations enriched in our study showed the potential to consume energy-rich complex carbon compounds including proteins, polysaccharides, nucleic acids, and lipids. These populations can mineralize the organic matter from primary production in overlying waters and from local chemosynthesis in hydrothermal sediments. Beyond Guaymas Basin, macromolecule-degrading thermophiles apparently occur more broadly in hydrothermal systems worldwide and are not limited to sediments but could thrive in hydrothermal chimneys or in surface-attached biofilms. The degradation of primary produced biomass or of necromass by uncultured heterotrophs may be a widespread process relevant for our understanding of the diversity and carbon cycling in marine ecosystems. Future studies are needed to characterize the abundance and activity of uncultured macromolecule degraders at cold seeps and hydrothermal vents to further improve our understanding of carbon exchanges between the lithosphere and biosphere.

## Supplementary information

Supplementary Information

Supplementary Data 1

Supplementary Data 2

Supplementary Data 3

Supplementary Data 4

Supplementary Data 5

Supplementary Data 6

Supplementary Data 7

## Data Availability

All sequence data is available at NCBI under BioProject ID PRJNA635695. Accession numbers for individual metagenomes can be found in Supplementary Information (Table S6).
