# Supplementary material for: Degradation of biological macromolecules supports uncultured microbial populations in Guaymas Basin hydrothermal sediments
Source: ISME J. 2021 Jun 10;15(12):3480–97. doi: 10.1038/s41396-021-01026-5 (PMC8630151; doi:10.1038/s41396-021-01026-5)
Supplement: Supplementary file 1 — Supplementary Information [file 41396_2021_1026_MOESM1_ESM.docx]

Supplementary Material for:

**Degradation of biological macromolecules supports uncultured microbial populations in Guaymas Basin hydrothermal sediments**

Sherlynette Pérez Castro; Borton, Mikayla A.; Regan, Kathleen; Hrabe de Angelis, Isabella; Wrighton, Kelly C.; Teske, Andreas P.; Strous, Marc; Ruff, S. Emil

Table of Contents

Shipment and of samples and preparation of slurries

Determination of optimal sample volume for inoculation

Media composition and preparation

Calibration of chromatograph and headspace measurement

Cell counts

Primer sequences

16S rRNA sequence processing

16S rRNA-based community analyses

Metagenomic sequencing, assembly, binning and analyses

Estimate of growth rate and biomass increase

Catabolic potential of polysaccharides degraders

Supplementary Table 1. Media composition

Supplementary Table 2. Summary of P-values for the linear mixed model for effects of carbon sources, redox conditions, sediment core, timepoint and the interaction on cell numbers and hydrogen gas.

Supplementary Table 3. Summary of P-values for the multiple comparisons of means (Tukey Contrasts) for effects of carbon sources on the in the production of $H_{2}$.

Supplementary Table 4. Summary of P-values for the pairwise comparisons using permutation MANOVAs on a distance matrix for effects of carbon sources and redox conditions on 16S rRNA genus-level bacterial relative sequence abundance.

Supplementary Table 5. MAGs classification as a potential polysaccharide degrader (black) or not (red) based on the number of enzymes involved in extracellular cleavage, transmembrane import, and intracellular degradation of polysaccharides (sum = extracellular CAZymes + CAZymes + transporters).

Supplementary Table 6. MAGs accession numbers

Supplementary Figures

**Shipment and of samples and preparation of slurries.**

After retrieval and sectioning of the sediment cores the samples were transferred to 250 ml wide-mouth screw-cap glass bottles and sterile artificial seawater (Red Sea Salt, 33.4 g l^-1^; http://www.redseafish.com) was added to exclude headspace and prevent the sediment from drying. After shipment to the laboratory at 4°C the sediment samples had a thin oxidized surface layer, yet the bulk sediments were still black and smelled sulfidic, showing that the samples’ redox state was not compromised. Within few days the experiment was set-up and 1:10 slurries (sediment: anoxic artificial seawater) were prepared for inoculation. For each sample (slurry) we used three sediment horizons, 0-2, 3-4 and 5-10 cm at a volume ratio of 1:1:3, respectively, to increase the diversity of sampled communities and include sediment layers that experience different temperature regimes from 3°C to above 50°C.

**Determination of optimal sample volume for inoculation.**

To achieve a good enrichment efficiency, we determined the number of cells that were needed to inoculate the cultures. To make sure that the cultures relied on the added carbon instead of residual carbon that was present in the inoculum, the added carbon needed to be an order of magnitude higher than the residual carbon. Because we were studying cellular polymer degradation, we estimated that the cells of the inoculum themselves were the residual carbon source of concern. We thus prepared the cultures so that the added carbon source contained at least 10 times more carbon than the cells present in the inocula. To provide realistic conditions we added biopolymers as sole carbon and energy source to gain a final concentration of 1 mM added carbon. To gain the desired enrichment efficiency, this meant we had to use an amount of inoculum that contained at most 0.1 mM carbon in the form of microbial cells.

To estimate the necessary cell number we used the following approach: we calculated the amount of carbon that we added to each culture (Cadded) when using 1 mM carbon. We then used the estimated biomass yield per gram glucose (Ybiomass) (Postma et al., 1989; Verduyn et al., 1990; Zeng et al., 1990) to determine the amount of biomass carbon (Cbiomass) that can be produced from the added carbon. This biomass carbon was then divided by the amount of carbon per single microbial cell (Ccell) (Fagerbakke et al., 1996) to obtain the number of cells (Ncells) that can be produced by the added carbon. We added an amount of inoculum that contained ten times less cells than Ncells to achieve a good enrichment efficiency:

Cadded [mg] = (MWCarbon [mg/mM] * Cconc [mM]) / (1000 [ml] / VCulture [ml])

Ybiomass = 0.2 g/g (anaerobes); 0.5 g/g (aerobes)

Cbiomass [mg] = Cadded [mg] * Ybiomass

Ccell [mg] = 25 * 10^-12^ g

Ncells = (Cbiomass / Ccell)

**Media composition and preparation.**

We used basal saltwater medium with sulfate for sulfate-reducing and oxic cultures and basal saltwater medium lacking sulfate (which was replaced by equimolar NaCl) for fermentative cultures (Widdel and Bak, 1992). Oxygen was removed from the media by purging the headspace three times with Argon, each time evacuating the bottles to a stable vacuum at ~50 mbar, followed by 3 bar over-pressure with Argon. To remove any remaining oxygen and other oxidants, we added 1 mM sodium sulfide to the medium, as recommended for anoxic media (Widdel and Bak, 1992). Carbon sources were used at a carbon equivalent of 1 mM. All media components are listed in Table S1.

**Calibration of chromatograph and headspace measurement.**

For each analysis we withdrew 250 µl of headspace using a gas-tight borosilicate glass syringe. The sample was injected into the instrument with argon as carrier gas. At the beginning of each measurement session, the instrument was calibrated by making a standard curve obtained from four samples of hydrogen or methane calibration gas at defined concentrations (500 ppm, 1000 ppm, 10000 ppm, 100000 ppm). Hydrogen or methane peak area was converted into mM using standard conversion factors.

**Cell counts.**

To fix cells we added 900 µl sample to 100 µl 37 % aqueous paraformaldehyde solution (f.c. 3.7 %). The fixed cells were sonicated at 21 % intensity for 20 seconds (Sonics VibraCell ultrasonic 100 W with microprobe). This step dislodged cells from sediments and reduced the size and thus interference of auto-fluorescing aggregates. Samples were diluted 1:1 with autoclaved MilliQ water. Ten µl diluted sample were mixed with 1 × PBS and filtered onto a polycarbonate filter (GTTP, 0.2 µm poresize, Millipore). Filter sections were incubated with 4',6-diamidino-2-phenylindole (DAPI, 1 µg ml^-1^) for 10 min at RT, rinsed with ultrapure water and with 80 % ethanol, and air dried. Filter sections were embedded on a microscopy slide in mounting medium (4:1 Citifluor:Vectashield) and enumerated using a Zeiss Axiolab epifluorescence microscope. Twenty grids (100 µm × 100 µm) per sample were counted.

**Primer sequences.**

The bacterial 16S rRNA gene V4-V5 variable region was amplified using the forward primer 518F (3’-CCAGCAGCYGCGGTAAN-5’) and reverse primers 926R (3’-CCGTCAATTCNTTTRAGT-5’, 3’-CCGTCAATTTCTTTGAGT-5’, 3’-CCGTCTATTCCTTTGANT-5’) (Nelson et al., 2014). The archaeal 16S rRNA gene V4-V5 variable region was amplified using the forward primers 517F (3’-GCCTAAAGCATCCGTAGC-5’, 3’-GCCTAAARCGTYCGTAGC-5’, 3’-GTCTAAAGGGTCYGTAGC-5’, 3’-GCTTAAAGNGTYCGTAGC-5’, 3’-GTCTAAARCGYYCGTAGC-5’) and reverse primer 958R (3’-CCGGCGTTGANTCCAATT) (Topçuoğlu et al., 2016).

**16S rRNA sequence processing.**

Raw sequences were processed following the DADA2 Pipeline Tutorial v1 (<https://benjjneb.github.io/dada2/tutorial.html>). Briefly, forward and reverse reads were quality-trimmed to 275 bp and 205 bp, respectively, and primer sequences (17 bp forward, 18 bp reverse) were removed. Reads with more than two expected errors were discarded, paired reads were merged, and chimeric sequences were removed. Amplicon sequence variants (ASVs) affiliating with Salmonella were derived from the added nucleic acid carbon source and were removed prior to the analyses. Species level taxonomy was assigned with the silva_nr_v132_train_set and silva_species_assignment_v132 based on the Silva small subunit reference database SSURef v132 (Quast et al., 2013).

**16S rRNA-based community analyses**.

To analyze and visualize bacterial and archaeal community structure, we used VisuaR (https://github.com/EmilRuff/VisuaR), a workflow based on custom scripts and various R packages including *vegan* v2.5-6 (Oksanen et al., 2019) and *ggplot2* v3.3.2 (Wickham, 2009) in R version 3.6.2. Shannon’s entropy was calculated based on relative abundance of ASVs*.* Bray-Curtis dissimilarities between relative sequence abundance at the genus level across all samples were compared by permutational multivariate analysis of variance (PerMANOVA) using the *adonis* function of the *vegan* package. When significant effects were found, multiple comparisons between culture conditions were performed using the pairwise.perm.manova function within the *RVAideMemoire* package (Hervé, 2020). Non-metric multidimensional scaling (NMDS) analysis was also used to visualize community similarity between culture conditions using the function *metaMDS* in the *vegan* package. Component scores were used to generate 95 % confidence ellipses around samples using the *ordiellipse* function in *vegan*.

**Metagenomic sequencing, assembly, binning, and analyses*.***

Quality control of the raw reads was performed using Preprocessing and Information of SEQuence data (PRINSEQ) to remove reads with less than 60 bp, mean quality score lower than 20, and with duplicates and N’s (Schmieder and Edwards, 2011). Read ends with quality score lower than 20 were also trimmed. All samples combined provided a total of ~130 megabases of sequencing data. All forward and reverse reads were placed together in one file and co-assembled using SPAdes with the *--meta* option (Bankevich et al., 2012). Co-assembly was performed to pool enough reads to reconstruct genomes belonging to the same environment. Binning was performed using MetaWRAP processing modules with initial extraction using MaxBin2, metaBAT2, and CONCOCT, bin refinement and reassembly (Uritskiy et al., 2018). Completeness and contamination of bins was assessed using CheckM (Parks et al., 2015). Assembled genomes that were of substantial quality as defined by containing more than 90 % genome completeness, less than 3 % contamination, and less than 100 contigs were further analyzed (Table 1). Taxonomy and closest phylogenetic neighbors were assigned using the GTDB-tk (v1.3.0) (Chaumeil et al., 2020), average nucleotide identity (ANI), average amino acid identity (AAI), and single-copy genes (Supplementary Data 1). Average Nucleotide Identity (ANI) and Average Amino Acid Identity (AAI) among genomes were calculated using EZBioCloud OrthoANIu Calculator (Yoon et al., 2017) (<https://www.ezbiocloud.net/tools/ani>) and Kostas Lab AAI calculator (aai.rb) (Konstantinidis and Tiedje, 2005), respectively. ANI values < 95 % were used as a conservative threshold for new species. AAI > 65 %, and > 45 % were used for the same genus and family, respectively (Konstantinidis et al., 2017).

**Estimate of growth rate and biomass increase using cell abundance.**

Using the parameters described in the previous section we can estimate how much carbon was consumed to produce a given increase in cell abundance. To estimate this increase in community biomass we take the difference in cell abundance (dNcells) between timepoint 3 (NcellsT3) and timepoint 1 (NcellsT1). This differential cell abundance (dNcells) is now multiplied by the amount of carbon per cell (Ccell) to obtain the total amount of carbon bound in the new cells (Cnewcells). The carbon in these new cells (Cnewcells) can then be related to the carbon that was added to the enrichments (Cadded) to estimate how much of the added carbon was transformed into new biomass (Cnewbiomass). If we consider the biomass yield (Ybiomass) - i.e. that only 20 % of the consumed carbon is used to make new biomass, while 80 % are respired for maintenance - we can also estimate how much carbon was needed to produce these new cells (Cnewcells+). This total amount of carbon used to produce new cells can be related again to the carbon added to obtain an understanding of the percentage of carbon that was used to produce the observed increase in community size (Cnewbiomass+).

dNcells = NcellsT3 - NcellsT1

Cnewcells [mg] = dNcells * Ccell [mg]

Cnewcells+ [mg] = Cnewcells [mg] / Ybiomass

(Cnewbiomass) * 100 [%] = Cnewcells [mg] / Cadded [mg] --> Percent of added carbon in new biomass

(Cnewbiomass+) * 100 [%] = Cnewcells+ [mg] / Cadded [mg] --> Percent of added carbon needed to produce new biomass

**Catabolic potential of polysaccharides degraders**.

We tested whether a MAG assigned as polysaccharide degrader contained more genes for the degradation of polysaccharides by comparing the number of enzymes involved in extracellular cleavage, transmembrane import, and intracellular degradation between MAGs enriched in polysaccharides-amended cultures and non-polysaccharides amended cultures (2 groups, Fig. 7, Table S5). We depicted the distribution of this parameter as a boxplot and a t-test was used to compare the two groups (Fig. S6).

**Supplementary Table 1.** Media composition

| **Mineral salt solution (per liter) - modified after Widdel and Bak 1992** | | |  |  |
| --- | --- | --- | --- | --- |
|  |  |  |  |  |
| Component | Amount (g) | MW | Final Conc. | 10X Amount (g) |
| NaCl | 20 | 58.44 | 342 mM | 200 |
| MgCl_2_ * 6 H_2_O | 3 | 203.3 | 14.8 mM | 30 |
| CaCl_2_ * 2 H_2_O | 0.15 | 147.02 | 1.0 mM | 1.5 |
| KCl | 0.5 | 74.56 | 6.71 mM | 5 |
| NH_4_Cl | 0.0135 | 53.49 | 0.25 mM | 0.135 |
|  |  |  |  |  |
| **Sulfate solution (per liter) for oxic and anoxic sulfate media** | | |  |  |
|  |  |  |  |  |
| Component | Amount (g) | MW | Final Conc. | 10X Amount (g) |
| Na_2_SO_4_ | 4 | 142 | 28 mM | 40 |
|  |  |  |  |  |
| **NaCl solution (per liter) for anoxic unamended media** | |  |  |  |
|  |  |  |  |  |
| Component | Amount (g) | MW | Final Conc. | 10X Amount (g) |
| NaCl | 1.6 | 58.44 | 28 mM | 16 |
|  |  |  |  |  |
| **Carbonate solution (per liter)** |  |  |  |  |
|  |  |  |  |  |
| Component | Amount (g) | MW | Final Conc. | 10X Amount (g) |
| NaHCO₃ | 0.084 | 84 | 1 mM | 0.84 |
|  |  |  |  |  |
| **Sodium phosphate buffer pH 7.2 (per liter)** | |  |  |  |
|  |  |  |  |  |
| Component | Amount (ml) of a 1 M stock | MW | Final Conc. | 100X Conc. |
| NaH_2_PO_4_/ Na_2_HPO_4_ | 31.6/68.4 ad 1l | 120/142 | 1 mM | 100 mM |
|  |  |  |  |  |
| **1000X HCl-Dissolved Trace Elements Stock Solution (per liter) - Widdel et al. 1983** | | | |  |
|  |  |  |  |  |
| Component | Amount | FW | 1000X Conc. | Final Conc. |
| HCl (37%) | 8.5 ml | na | 100 mM | 100 μM |
| FeSO_4_ * 7 H_2_O | 2100 mg | 278.01 | 7.5 mM | 7.5 μM |
| H_3_BO_3_ | 30 mg | 61.83 | 0.48 mM | 0.48 μM |
| MnCl₂ * 4 H_2_O | 100 mg | 197.91 | 0.5 mM | 0.5 μM |
| CoCl_2_ * 6 H_2_O | 190 mg | 237.93 | 6.8 mM | 6.8 μM |
| NiCl₂ * 6 H_2_O | 24 mg | 237.69 | 1.0 mM | 1.0 μM |
| CuCl_2_ * 2 H_2_O | 2 mg | 170.48 | 12 μM | 12 nM |
| ZnSO₄ * 7 H_2_O | 144 mg | 287.56 | 0.5 mM | 0.5 μM |
| Na_2_MoO_4_ * 2 H_2_O | 36 mg | 241.95 | 0.15 mM | 0.15 μM |
|  |  |  |  |  |
| **1000X NaOH-Dissolved Trace Elements Stock Solution (per liter) - Widdel et al. 1983** | | | |  |
|  |  |  |  |  |
| Component | Amount | FW | 1000X Conc. | Final Conc. |
| NaOH | 400 mg | - | - | - |
| Na_2_WO_4_ * 2 H_2_O | 6 mg | 329.85 | 18 μM | 18 nM |
| Na_2_SeO_3_ * 5 H_2_O | 8 mg | 263.01 | 32 μM | 32 nM |
|  |  |  |  |  |
| **Carbon source (per 250 ml) - only one carbon source was used for each medium** | | | |  |
|  |  |  |  |  |
| Component | Amount | MW (monomer) | Stock Conc. | Final Conc. |
| BSA | 250 mg | 114 | 40 X | 1 C mM |
| Nucleotides | 33 mg | 327 | 4 X | 1 C mM |
| L-Phosphatidylcholine | 180 mg | 775 | 40 X | 1 C mM |
| Polysaccharides (mix) | 300 mg | 180 | 40 X | 1 C mM |

**Supplementary Table 2.** Summary of P-values for the linear mixed model for effects of carbon sources, redox conditions, sediment core, timepoint and the interaction on cell numbers and hydrogen gas.

| Factor(s) | Cell numbers | Hydrogen gas |
| --- | --- | --- |
| Carbon source | 0.002 | <0.0001 |
| Redox Condition | 0.61 | <0.0001 |
| Sediment Core | NA | 0.0003 |
| Timepoint | 0.03 | 0.03 |
| Carbon:Redox | 0.29 | <0.0001 |
| Carbon:Core | NA | 0.001 |
| Electron:Core | NA | 0.001 |
| Carbon:Time | 0.71 | 0.02 |
| Redox:Time | 0.74 | 0.03 |
| Core:Time | NA | 0.06 |
| Carbon:Redox:Core | NA | 0.0001 |
| Carbon:Redox:Time | 0.48 | 0.58 |
| Carbon:Core:Time | NA | 0.003 |
| Redox:Core:Time | NA | 0.70 |
| Carbon:Electron:Core:Time | NA | 0.38 |

**Supplementary Table 3.** Summary of P-values for the multiple comparisons of means (Tukey Contrasts) for effects of carbon sources on the in the production of $H_{2}$.

| DNA | - | PRO | 0.99527 |
| --- | --- | --- | --- |
| LIP | - | PRO | <0.001 |
| POL | - | PRO | 0.00146 |
| LIP | - | DNA | <0.001 |
| POL | - | DNA | 0.004 |
| POL | - | LIP | 0.69719 |

**Supplementary Table 4.** Summary of P-values for the pairwise comparisons using permutation MANOVAs on a distance matrix for effects of carbon sources and redox conditions on 16S rRNA genus-level bacterial relative sequence abundance.

|  | PRO | DNA | LIP |
| --- | --- | --- | --- |
| DNA | 0.150 | - | - |
| LIP | 0.078 | 0.078 | - |
| POL | 0.085 | 0.085 | 0.078 |

|  | oxic | sulfate-amended |
| --- | --- | --- |
| sulfate-amended | 0.0045 | - |
| fermentative | 0.003 | 0.689 |

**Supplementary Table 5.** MAGs classification as a potential polysaccharide degrader (black) or not (red) based on the number of enzymes involved in extracellular cleavage, transmembrane import, and intracellular degradation of polysaccharides (sum = extracellular CAZymes + CAZymes + transporters, Fig. S6).

| bin | CPM | extraCAZymes | CAZymes | transporters | sum |
| --- | --- | --- | --- | --- | --- |
| Bacillus GB_116 | 1581 | 2 | 20 | 2 | 24 |
| UBA2242 GB_043 | 761 | 6 | 101 | 0 | 107 |
| Kosmotoga GB_055 | 212 | 2 | 35 | 6 | 43 |
| Thermococcus GB_027 | 111 | 0 | 23 | 2 | 25 |
| UBA2242 GB_103 | 104 | 3 | 46 | 2 | 51 |
| Desulforudaceae GB_121 | 73 | 0 | 16 | 0 | 16 |
| UBA5301 GB_067 | 7 | 0 | 18 | 2 | 20 |
| Thermosipho GB_064 | 5 | 1 | 33 | 7 | 41 |
| Thermovirga GB_013 | 2 | 0 | 6 | 0 | 6 |
| Brevibacillus GB_061 | 0 | 3 | 34 | 2 | 39 |
| Thermosipho GB_058 | 0 | 2 | 32 | 5 | 39 |
| Alkalibacillaceae GB_076 | 0 | 2 | 22 | 2 | 26 |
| Brevibacillaceae GB_007 | 0 | 3 | 20 | 2 | 25 |
| ZCTH02-B6 GB_124 | 0 | 0 | 19 | 3 | 22 |
| Calderihabitantaceae GB_131 | 0 | 1 | 19 | 0 | 20 |
| Desulfutomaculum GB_002 | 0 | 0 | 11 | 2 | 13 |
| Thermosyntropha GB_081 | 0 | 0 | 11 | 0 | 11 |
| Thermodesulfobacterium GB_111 | 0 | 0 | 9 | 2 | 11 |
| Archaeoglobus GB_049 | 0 | 0 | 9 | 0 | 9 |
| Archaeoglobus GB_100 | 0 | 0 | 3 | 0 | 3 |

**Supplementary Table 6.** Accession numbers for all metagenome assembled genomes binned from Guaymas Basin thermophilic enrichment cultures available at NCBI under BioProject ID PRJNA635695.

| NCBI accession | NCBI sample_name | | Organism | Tax ID |
| --- | --- | --- | --- | --- |
| SAMN15049706 | | GB_MAG1_027 | Thermococcus sp. | 35749 |
| SAMN15049707 | | GB_MAG2_100 | Archaeoglobus sp. | 1872626 |
| SAMN15049708 | | GB_MAG3_049 | Archaeoglobus sp. | 1872626 |
| SAMN15049709 | | GB_MAG4_043 | Candidatus Marinimicrobia bacterium UBA2242 | 1953229 |
| SAMN15049710 | | GB_MAG5_103 | Candidatus Marinimicrobia bacterium UBA2242 | 1953229 |
| SAMN15049711 | | GB_MAG6_111 | Thermodesulfobacterium sp. | 1965289 |
| SAMN15049712 | | GB_MAG7_055 | Kosmotoga sp. | 1955248 |
| SAMN15049713 | | GB_MAG8_064 | Thermosipho sp. | 1968895 |
| SAMN15049714 | | GB_MAG9_058 | Thermosipho sp. | 1968895 |
| SAMN15049715 | | GB_MAG10_013 | Thermovirga sp. | 2699834 |
| SAMN15049716 | | GB_MAG11_116 | Bacillus sp. | 1409 |
| SAMN15049717 | | GB_MAG12_076 | Bacillaceae bacterium | 1889774 |
| SAMN15049718 | | GB_MAG13_007 | Bacillaceae bacterium | 1889774 |
| SAMN15049719 | | GB_MAG14_061 | Brevibacillus sp. | 1882945 |
| SAMN15049720 | | GB_MAG15_131 | Thermoanaerobacteraceae bacterium | 2100788 |
| SAMN15049721 | | GB_MAG16_081 | Thermosyntropha sp. | 2740820 |
| SAMN15049722 | | GB_MAG17_124 | Firmicutes bacterium | 1879010 |
| SAMN15049723 | | GB_MAG18_067 | Firmicutes bacterium UBA5301 | 1948048 |
| SAMN15049724 | | GB_MAG19_121 | Peptococcaceae bacterium | 2052179 |
| SAMN15049725 | | GB_MAG20_002 | Desulfotomaculum sp. | 41211 |

**Supplementary Figures**

**a**

**b**

**

**Supplementary Figure 1.** a) Cell numbers in inocula and enrichment cultures during week 1 and 3. b) Cell numbers versus concentration of hydrogen in enrichment cultures during week 3.

**Supplementary Figure 2.** Non-metric multidimensional scaling (NMDS) analyses (95 % confidence ellipse) in enrichment cultures supplemented with proteins (PRO), nucleic acids (DNA), lipids (LIP) or polysaccharides (POL) under oxic, sulfate-reducing or fermentative (no added electron acceptor) conditions after four weeks of incubation. Relative sequence abundances are based on 16S rRNA V4/V5 gene amplicons.

**Supplementary Figure 3.** Relative sequence abundance of the most abundant bacterial genus-level clades in in enrichment cultures supplemented with proteins (PRO), nucleic acids (DNA), lipids (LIP) or polysaccharides (POL) under aerobic, sulfate-reducing, or fermentative (no added electron acceptor) conditions after four weeks of incubation. The used inoculum is indicated at the top of the graph. Relative sequence abundances are based on 16S rRNA V4/V5 gene amplicons.

**Supplementary Figure 4.** Relative sequence abundance of the most abundant archaeal genera found in cultures supplemented with proteins (BSA), nucleic acids (DNA), lipids (LIP) and polysaccharides (POL) under sulfate-reducing and fermentative conditions (Una) and in the hot sediment that served as inoculum. Based on 16S rRNA V4/V5 regions surveys. Uncultured genera are shown by the prefix “unc_” and the closest lineage with cultured representatives. E.g., unc_Thermoproteales represents an uncultured genus within the order *Thermoproteales*.

**Supplementary Figure 5.** Glycoside hydrolase families (GH), polysaccharide lyases (PL), selected pathways and transporters found in *Marinisomatota* UBA2242 MAGs GB043 (blue), or GB103 (red), or both (pink). The compounds listed in brackets feature glycosyl bonds that match with the specificity of the respective CAZyme, potentially representing substrates.

**Supplementary Figure 6.** Boxplot representing the median and one quartile deviation of MAGs potential to degrade polysaccharides based on the number of enzymes involved in extracellular cleavage, transmembrane import, and intracellular degradation of the macromolecule.

**References**

Bankevich, A., Nurk, S., Antipov, D., Gurevich, A.A., Dvorkin, M., Kulikov, A.S., Lesin, V.M., Nikolenko, S.I., Pham, S., Prjibelski, A.D., Pyshkin, A. V., Sirotkin, A. V., Vyahhi, N., Tesler, G., Alekseyev, M.A., Pevzner, P.A., 2012. SPAdes: A New Genome Assembly Algorithm and Its Applications to Single-Cell Sequencing. Journal of Computational Biology 19, 455–477. doi:10.1089/cmb.2012.0021

Bowers, R.M., Kyrpides, N., Stepanauskas, R., Harmon-Smith, M., Doud, D., Reddy, T., Schulz, F., Jarett, J., Rivers, A.R., Eloe-Fadrosh, E., Tringe, S.G., Ivanova, N.N., Copeland, A., Clum, A., Becraft, E.D., Malmstrom, R.R., Birren, B., 2017. Minimum information about a single amplified genome (MISAG) and a metagenome-assembled genome (MIMAG) of bacteria and archaea. Nature Biotechnology 35, 725–731. doi:10.1038/nbt.3893

Chaumeil, P.A., Mussig, A.J., Hugenholtz, P., Parks, D.H., 2020. GTDB-Tk: A toolkit to classify genomes with the genome taxonomy database. Bioinformatics 36, 1925–1927. doi:10.1093/bioinformatics/btz848

Fagerbakke, K.M., Heldal, M., Norland, S., 1996. Content of carbon, nitrogen, oxygen, sulfur and phosphorus in native aquatic and cultured bacteria. Aquatic Microbial Ecology 10, 15–27.

Hervé, M., 2020. Testing and Plotting Procedures for Biostatistics.

Konstantinidis, K.T., Rosselló-Móra, R., Amann, R., 2017. Uncultivated microbes in need of their own taxonomy. ISME Journal 11, 2399–2406. doi:10.1038/ismej.2017.113

Konstantinidis, K.T., Tiedje, J.M., 2005. Towards a genome-based taxonomy for prokaryotes. Journal of Bacteriology 187, 6258–6264. doi:10.1128/JB.187.18.6258-6264.2005

Nelson, M.C., Morrison, H.G., Benjamino, J., Grim, S.L., Graf, J., 2014. Analysis, optimization and verification of illumina-generated 16s rRNA gene amplicon surveys. PLoS ONE 9. doi:10.1371/journal.pone.0094249

Oksanen, J., Blanchet, F.G., Friendly, M., Kindt, R., Legendre, P., Mcglinn, D., Minchin, P.R., O’Hara, R.B., Simpson, G.L., Solymos, P., Stevens, M.H.H., Szoecs, E., Wagner, H., 2019. Community Ecology Package.

Parks, D.H., Imelfort, M., Skennerton, C.T., Hugenholtz, P., Tyson, G.W., 2015. CheckM: assessing the quality of microbial genomes recovered from isolates, single cells, and metagenomes. Genome Research 25, 1043–1055. doi:10.1101/gr.186072.114

Postma, E., Verduyn, C., Scheffers, W.A., Van Dijken, J.P., 1989. Enzymic analysis of the crabtree effect in glucose-limited chemostat cultures of Saccharomyces cerevisiae. Applied and Environmental Microbiology 55, 468–477. doi:10.1128/aem.55.2.468-477.1989

Quast, C., Pruesse, E., Yilmaz, P., Gerken, J., Schweer, T., Yarza, P., Peplies, J., Glöckner, F.O., 2013. The SILVA ribosomal RNA gene database project: improved data processing and web-based tools. Nucleic Acids Research 41, D590–D596. doi:10.1093/nar/gks1219

Schmieder, R., Edwards, R., 2011. Quality control and preprocessing of metagenomic datasets. Bioinformatics (Oxford, England) 27, 863–4. doi:10.1093/bioinformatics/btr026

Topçuoğlu, B.D., Stewart, L.C., Morrison, H.G., Butterfield, D.A., Huber, J.A., Holden, J.F., 2016. Hydrogen Limitation and Syntrophic Growth among Natural Assemblages of Thermophilic Methanogens at Deep-sea Hydrothermal Vents. Frontiers in Microbiology 7, 1–12. doi:10.3389/fmicb.2016.01240

Uritskiy, G. V., Diruggiero, J., Taylor, J., 2018. MetaWRAP - A flexible pipeline for genome-resolved metagenomic data analysis 08 Information and Computing Sciences 0803 Computer Software 08 Information and Computing Sciences 0806 Information Systems. Microbiome 6, 1–13. doi:10.1186/s40168-018-0541-1

Verduyn, C., Postma, E., Scaffers, A., van Dijken, J.P., 1990. Energetics of Saccharomyces cerevisiae in anaerbic glucose-limited chemostat cultures. Journal of General Microbiology 136, 405–412.

Wickham, H., 2009. ggplot2: Elegant Graphics for Data Analysis. Springer New York.

Widdel, F., Bak, F., 1992. Gram-Negative Mesophilic Sulfate-Reducing Bacteria, in: Balows, A., Trüper, H.G., Dworkin, M., Harder, W., Schleifer, K.-H. (Eds.), The Prokaryotes: A Handbook on the Biology of Bacteria: Ecophysiology, Isolation, Identification, Applications. Springer New York, New York, NY, pp. 3352–3378. doi:10.1007/978-1-4757-2191-1_21

Yoon, S.-H., Ha, S., Lim, J., Kwon, S., Chun, J., 2017. A large-scale evaluation of algorithms to calculate average nucleotide identity. Antonie van Leeuwenhoek 110, 1281–1286. doi:10.1007/s10482-017-0844-4

Zeng, A.P., Ross, A., Deckwer, W.D., 1990. A method to estimate the efficiency of oxidative phosphorylation and biomass yield from ATP of a facultative anaerobe in continuous culture. Biotechnology and Bioengineering 36, 965–969. doi:10.1002/bit.260360912
